# Type III Interferon Induces Distinct SOCS1 Expression Pattern that Contributes to Delayed but Prolonged Activation of Jak/STAT Signaling Pathway: Implications for Treatment Non-Response in HCV Patients

**DOI:** 10.1371/journal.pone.0133800

**Published:** 2015-07-20

**Authors:** Bing Liu, Shan Chen, Yujuan Guan, Limin Chen

**Affiliations:** 1 The Institute of Blood Transfusion, Chinese Academy of Medical Sciences and Peking Union Medical College, Chengdu, Sichuan, People’s Republic of China; 2 Guangzhou No.8 People's Hospital, Guangzhou, China; 3 Toronto General Research Institute, University Health Network, University of Toronto, Toronto, Ontario, Canada; Temple University School of Medicine, UNITED STATES

## Abstract

Suppressor of cytokine signaling 1 (SOCS1) has long been thought to block type I interferon signaling. However, IFN-λ, a type III IFN with limited receptor expression in hepatic cells, efficiently inhibits HCV (Hepatitis C virus) replication in vivo with potentially less side effects than IFN-α. Previous studies demonstrated that type I and type III activated Janus kinase/signal transducer and activator of transcription (Jak/STAT) signaling pathway differently, with delayed but prolonged activation by IFN-λ stimulation compared to IFNα/β. However, the molecular mechanisms underlying this observation is not well understood. Here, we found that there are distinct differences in SOCS1 expression patterns in Huh-7.5.1 cells following stimulation with IFN-α and IFN-λ. IFN-λ induced a faster but shorter expression of SOCS1. Furthermore, we confirmed that SOCS1 over-expression abrogates anti-HCV effect of both IFN-α and IFN-λ, leading to increased HCV RNA replication in both HCV replicon cells and JFH1 HCV culture system. In line with this, SOCS1 over-expression inhibited STAT1 phosphorylation, attenuated IFN-stimulated response elements (ISRE) reporter activity, and blocked IFN-stimulated genes (ISGs) expression. Finally, we measured SOCS1 mRNA expression levels in peripheral blood mononuclear cells (PBMCs) with or without IFN-α treatment from 48 chronic hepatitis C patients and we found the baseline SOCS1 expression levels are higher in treatment non-responders than in responders before IFN-α treatment. Taken together, SOCS1 acts as a suppressor for both type I and type III IFNs and is negatively associated with sustained virological response (SVR) to IFN-based therapy in patients with HCV. More importantly, faster but shorter induction of SOCS1 by IFN-λ may contribute to delayed but prolonged activation of IFN signaling and ISG expression kinetics by type III IFN.

## Introduction

An estimated of 130–170 million people are chronically infected with HCV worldwide, which is a growing global pandemic and financial burden to the society [1]. With successful development of direct acting antiviral antivirals (DAAs), SVR in HCV chronically infected patients has been increased tremendously [2–4]. However, due to high cost, PEG-IFN/RBV is still the standard of care (SOC) to treat HCV infection in most developing countries [5]. Therefore, it is essential to develop methods to predict treatment response and uncover mechanism of viral resistance to further increase SVR. However, in consideration of viral resistance and side effects due to the fact that virtually all cell types express type I IFN receptor [6], other types of interferons with better efficacy and less toxicity should be explored. As a consequence, IFN-λs, which trigger the overlapping Jak/STAT signaling pathway with IFN-α through distinct receptors expressed only in restricted cell types [7], is an ideal therapeutic candidate for HCV therapy. Indeed, recombinant IFN-λ is currently being tested in clinical trial with promising preliminary data [8–10].

Although they signal through distinct receptors, signaling cascades of both IFN-α and IFN-λ converge on the activation of Janus kinase 1 (JAK1) and tyrosine kinase 2 (TYK2), leading to the subsequent phosphorylation and activation of latent signal transducer to form interferon stimulated gene factor 3 (ISGF3), that binds to the ISRE in the promoter regions and stimulates the expression of numerous ISGs [11–13]. The proteins encoded by these ISGs have different biological functions. Some mediate a myriad of antiviral activities, such as interferon stimulated gene 15 (ISG15) and myxovirus resistance-A (MxA) [14]. In order to prevent over-activation of the IFN signaling pathways, a few negative regulators are also induced following IFN stimulation, and SOCS1 is one of the classical inhibitors [15]. The SOCS protein family consists of eight members, which act as negative regulators of the IFN pathway, particularly SOCS1 [16]. SOCS1 protein contains an N-terminal region that includes the kinase inhibitory region (KIR), a central SH2 domain and a C-terminal SOCS box [17], which all is involved in inhibition of IFN signaling by means of interaction with Tyk2 [18].

In patients with chronic HCV infection, intrahepatic mRNA level of SOCS1 was associated with treatment outcomes following IFN-α therapy and degree of cirrhosis [19]. However, the relationship between SOCS1 expression level in PBMCs and response to therapy in patients with HCV remains unclear. Therefore, in the current study we analyzed whether endogenous SOCS1 was induced differently by exogenous IFN-α and IFN-λ in hepatic cell lines. We found that there was distinct differences in SOCS1 expression patterns in Huh-7.5.1 cells following IFN-α and IFN-λ stimulation. Although SOCS1 over-expression abrogated both type I and type III IFN anti-viral effect in JFH1 and HCV replicon model, type III IFN induced a faster but shorter expression of SOCS1, which may contribute to the delayed but prolonged activation of Jak/STAT signaling as reported in previous studies. Furthermore, SOCS1 expression in PBMCs is negatively associated with treatment outcomes (SVR) in patients chronically infected with HCV.

## Materials and Methods

### Cell culture and virus

Huh-7.5.1 cell line and Con1b subgenomic genotype 1b HCV replicon cell line were generously provided by Dr. Ian McGilvray (University of Toronto, Canada). The Con1b cell line is a Huh-7.5.1 cell population containing the subsequence genome HCV genotype 1b replicon. The cells were maintained in Dulbecco’s Modified Eagle’s Medium (DMEM) supplemented with 10% fetal bovine serum (FBS), antibiotics (100 units/mL penicillin and streptomycin), 100 μg/mL non-essential amino acid, and selection antibiotic G418 (1000 μg/mL) for Con1b cells. HCV infectious clone J6/JFH1was kindly provided by Dr. Charles Rice (Rockefeller University) as previously described [20].

### Clinical Samples

Forty-eight patients with chronic HCV (32 genotype 1, 2 genotype 1b, 5 genotype 6, and 9 unknown genotype) were treated at the Guangzhou No.8 People's Hospital. The demographic data of these patients are summarized in Table 1. All patients were treated with PEG-IFN/RBV for 48 weeks. Patients were designated as non-responders (NRs) if HCV RNA was detectable at the end of therapy, as relapses if HCV RNA was undetectable at the end of treatment but was detectable at the 6-month follow-up, and as having a sustained viral response if HCV RNA was undetectable at both the end of therapy and the 6-month follow-up. 26 individuals achieved sustained virological response and the remaining 22 patients were deemed non-responders. PBMCs were obtained from patients’ blood 24 weeks after the end of therapy and divided into two part. One part is cultured in vitro with IFN-α (500 IU/mL) treatment for 8 hours, whereas the other is cultured in vitro without IFN-α. Total RNA was isolated by Trizol method (described below). For SOCS1 detection, RNA was reverse transcribed to cDNA and subjected to real-time PCR as described below.

**Table 1:** Comparison of groups with and without sustained virological response.

	SVR (n = 26)	non-SVR (n = 22)
**Age; mean (range)**	46.6	54.5
**Male: female**	16:10	11:11
**HCV genotype**		
**1**	16	16
**1b**	2	0
**6**	3	2
**NA**	5	4

NA, not applicable; SVR, duration of treatment before achieving clinically diagnosed sustained virological response.

Written informed consent was obtained from each patient and the study protocol conformed to the ethics guidelines of the Declaration of Helsinki and was approved by the ethics review committees of Guangzhou No.8 People's Hospital.

### *In vitro* transfection and infection

The pCR3.1/SOCS1 construct was a gift from Professor Leiliang Zhang (Institute of Pathogen Biology, Chinese Academy of Medical Sciences and Peking Union Medical College). Cells were plated at 2×10^5^/mL, 500 uL per well onto a 24-well plate for 24 hours. Transfection with 4ug of pCR3.1 (blank vector/mock) and pCR3.1/SOCS1 using Fugene HD (Roche Diagnostics, Indianapolis, IN) according to the manufacturer’s protocol. IFN-α (Peg-interferon alfa-2b, Schering Co) and IFN-λ (IFN-lambda1 human recombinant, Sigma) was added to a final concentration of 100 IU/mL and 50 ng/mL for another 24 hours. Cells were harvested at 48 hours posttransfection and total RNA was extracted as described below. For Huh-7.5.1 cells infected with JFH1 inoculum (MOI = 0.56), RT-PCR were performed 48 hours after plasmid transfection, 54 hours JFH1 HCV infection, and 24 hours of IFN-α or IFN-λ treatment.

### RNA extraction and quantitative RT-PCR

Total RNA was isolated using Trizol method (Invitrogen, USA) according to the manufacturer’s instructions as previously described [20]. Total cDNA was synthesized by reverse transcription using First Strand cDNA Synthesis Kit (Bio-rad, USA) with random primers. The real-time RT-PCR for the quantification of HCV, ISG15, MxA, SOCS1, and glyceraldehyde-3-phosphate dehydrogenase (GAPDH) mRNAs were performed with the FastStart Universal SYBR Green Master Mix (Roche, USA). The primers used in this study were listed in S1 Table. The reaction mixture was first denatured at 95°C for 4 min and then 40 cycles of PCR were performed using the following protocol: 95°C 30 s; 60°C 30 s; 72°C 30 s. The mRNA level of each selected gene was normalized with GAPDH to obtain mRNA relative expression level (arbitrary unit).

### ISRE-luciferase reporter assay

ISRE signaling was monitored by the Promega dual-luciferase reporter assay system (Promega, Madison, WI) as previously described [21]. Briefly, the plasmid pISRE-luc (2 ug/mL) and pRL-TK (2 ng/mL) were cotransfected with pCR3.1 or pCR3.1/SOCS1 (4 ug/mL) in Con1b replicon cells using Fugene HD Transfection Reagent (Roche, Indianapolis, IN) following the manufacturer’s protocol. ISRE-mediated transcription was investigated in the presence of 100 IU/mL pegylated IFN-α or 50 ng/mL IFN-λ for 24 hours and relative luciferase activity was assessed.

### Protein sample preparation and Western blotting analysis

To harvest the protein lysates, cells were lysed using radioimmune precipitation assay (RIPA) buffer (Beyotime, China). Whole cell protein lysates were subsequently sonicated and boiled at 95°C for 5 min in SDS-PAGE sample buffer. Protein samples were separated by SDS-polyacrylamide gel electrophoresis (12% polyacrylamide gels) and transferred to PVDF membranes, and the membranes were blocked with 5% bovine serum albumin (BSA). The primary antibodies contained rabbit anti-MXA (Abcam, Cambridge, MA), rabbit anti-STAT1, rabbit anti-Phospho-STAT1 (Tyr701) (Cell Signaling Technology, Inc., Beverly, MA), rabbit anti-ISG15 antibodies (the kind gift of Dr. Ian McGilvray, University of Toronto, Canada), mouse anti-flag (Abcam, Cambridge, MA), and mouse anti-β-actin (Zhongshan Gold bridge, Beijing, China). The secondary antibodies were HRP-conjugated ECL goat anti-rabbit IgG, or HRP-conjugated ECL sheep anti-mouse IgG (Zhongshan Gold Bridge, Beijing, China). Blots were visualized with enhanced chemiluminescent detection reagents Immobilon Western Chemiluminesent HRP Substrate (Millipore, USA) on ImageQuant LAS 4000 mini (GE, USA).

### Statistical analyses

Statistical analysis was performed using Student's *t* test or one-way ANOVA and *p* values less than 0.05 were considered statistically significant. “*” means *p* values less than 0.05; “**” *p* values less than 0.01; “***” means *p* values less than 0.001. All data are representative of at least 3 repeated experiments.

## Results

### IFN-λ induced a faster but shorter SOCS1 expression than IFN-α which correlates with different ISG expression pattern in Huh-7.5.1 cells

**Fig 1:**
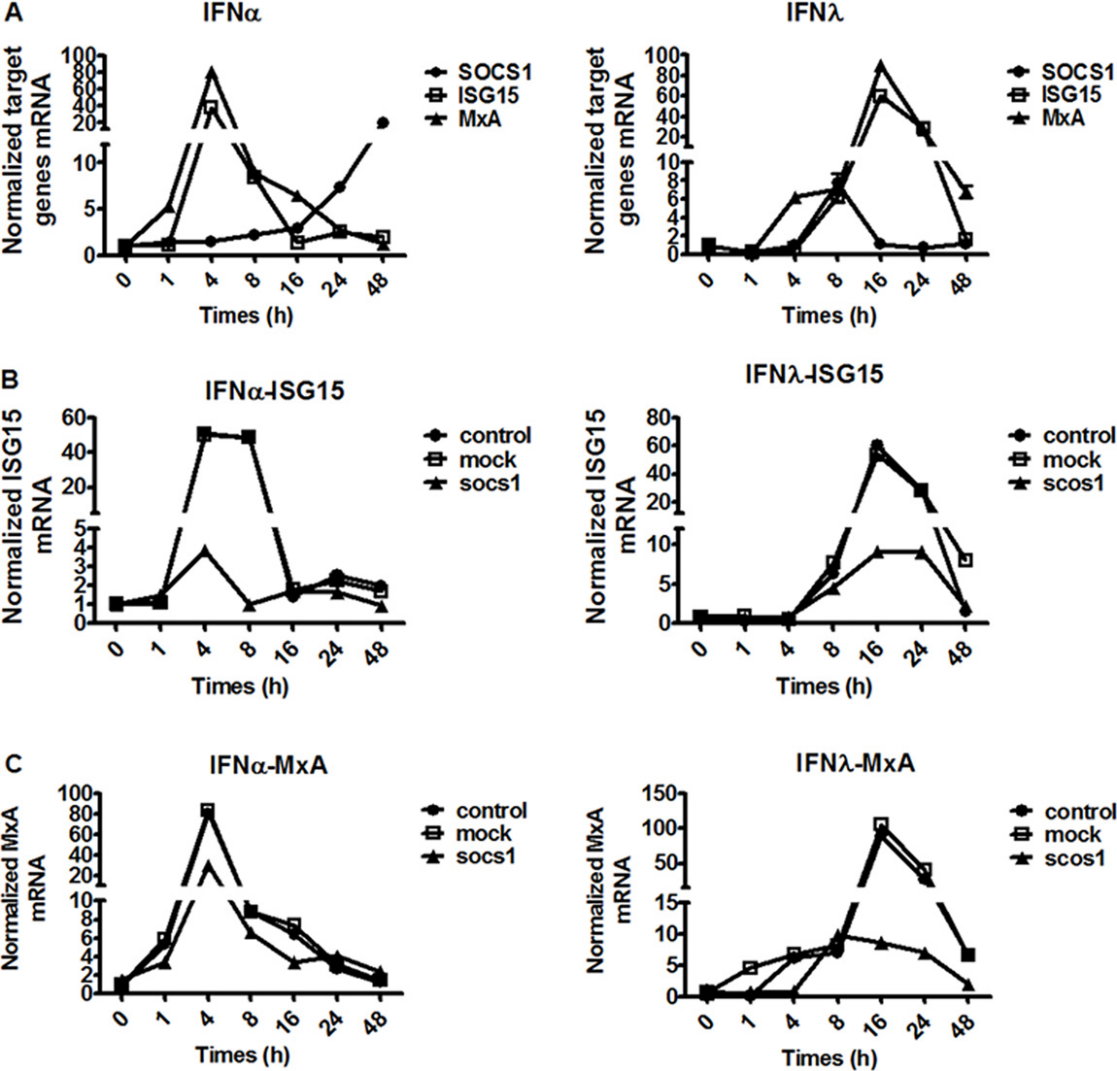
Different induction kinetics of ISGs (SOCS1, ISG15, MxA) by type I and type III IFNs. (A) Induction by 100 IU/mL IFN-α or 50 ng/mL IFN-λ for 0, 1, 4, 8, 16, 24 and 48 hours in Huh-7.5.1. (B) Induction kinetics of ISG15 after stimulation with 100 IU/mL IFN-α or 50 ng/mL IFN-λ in the absence and presence of SOCS1 over-expression. (C) Induction kinetics of MxA after stimulation with 100 IU/mL IFN-α or 50 ng/mL IFN-λ in the absence and presence of SOCS1 over-expression. Total RNA was harvested and reverse transcribed. The levels of mRNA expression were determined by quantitative real time PCR normalized to GAPDH. Data are presented as means ± SEM, n = 3. Error bars indicate standard error of mean (SEM).

To test whether SOCS1 is induced differently by type I and type III, we stimulated Huh-7.5.1 cells with 100 IU/mL IFN-α or 50 ng/mL IFN-λ for 0, 1, 4, 8, 16, 24 and 48 hours and SOCS1 and ISGs (ISG15 and MxA, two most commonly studied ISGs) mRNA expression levels were determined by quantitative real-time PCR. As shown in Fig 1A, there are distinct differences of SOCS1 expression patterns in Huh-7.5.1 cells upon stimulation with IFN-α or IFN-λ. While SOCS1 induction peaked at 8 hours and died down rapidly following stimulation with IFN-λ, IFN-α produced a prolonged induction pattern of SOCS1 expression, lasting for more than 48 hours. This differential induction pattern of SOCS1 is consistent with differential expression kinetics of ISGs following type I or type III IFN stimulation that IFN-λ induced a delayed but prolonged activation of ISG expression.

Having observed the different kinetic profiles of SOCS1 expression stimulated by IFN-α and IFN-λ, we subsequently investigated the role of SOCS1 over-expression on the expression kinetics of ISGs. This was accomplished by comparing ISG15 and MxA, kinetic patterns in the presence of either a SOCS1-expression vector or empty vector (mock). As predicted, IFN-α activated IFN signaling resulting in the increased expression of ISGs faster but lasted shorter compared to IFN-λ, and SOCS1 over-expression significantly blunted the increases expression of ISG15 following stimulation with IFN-α and IFN-λ (Fig 1B). Similar results were observed for MxA mRNA expression in the presence of SOCS1 upon stimulation with IFN-α and IFN-λ (Fig 1C).

### SOCS1 over-expression inhibits both type I and type III IFN signaling: inhibited STAT1 phosphorylation, attenuated ISRE reporter activity, and blocked ISGs expression

To monitor the role of SOCS1 over-expression in interferon-induced signaling pathway in cells, we analyzed phosphorylation of STAT1, the interferon stimulated response element (ISRE) activity and ISGs expressions level, which plays an essential role in IFN signaling pathway from up-stream to down-stream.

**Fig 2:**
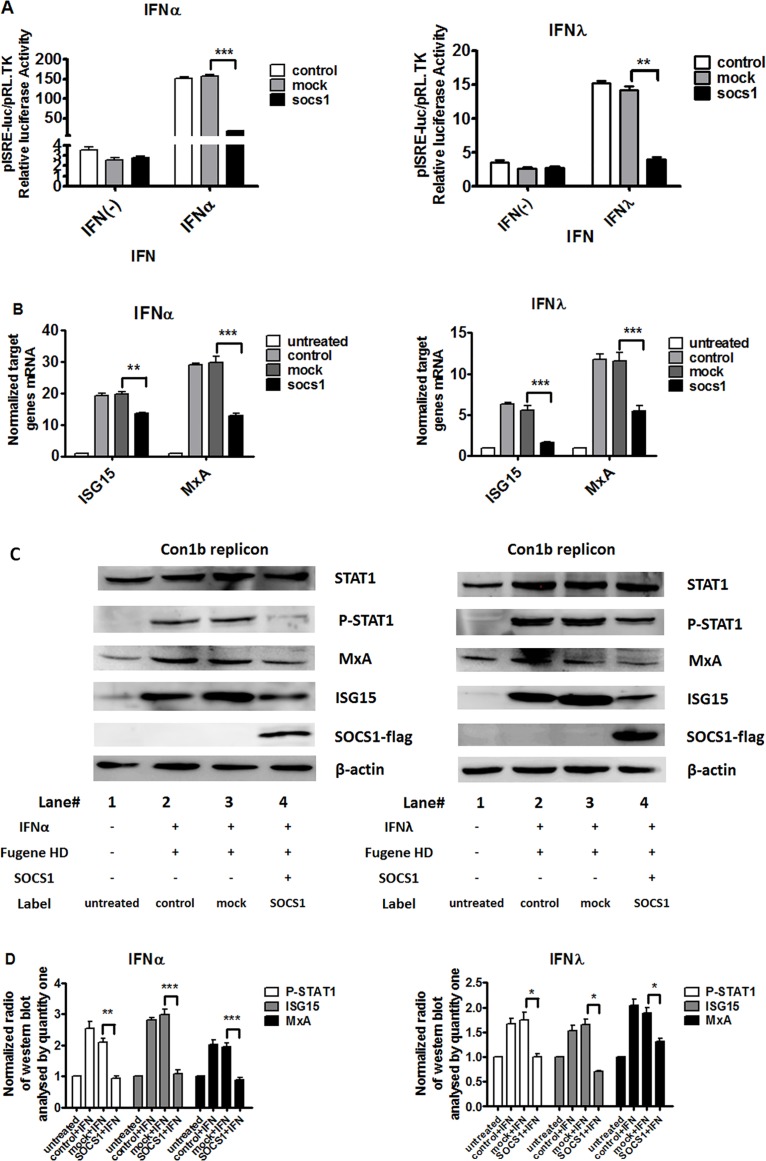
Over-expression of SOCS1 blocked IFN-α and IFN-λ signaling pathway. Over-expression of SOCS1 repressed IFN-induced ISRE-luciferase activity (A), decreased IFN-α and IFN-λ induced ISG15 and MxA (B) mRNA and protein levels (C) and STAT1 phosphorylation (C) in Con1b replicon cell. (D) Western blot data analyzed by Quantity One are expressed as the means of ratios of targeted genes (pSTAT1, ISG15 and MxA) /β-actin. Con1b cells were cotransfected with pCR3.1 (mock) or pCR3.1/SOCS1, pISRE-luc and pRL-TK for 24 hours and then 100 IU/mL IFN-α and 50 ng/mL IFN-λ was added to the cells for 24 hours respectively. The firefly and Renilla luciferase activity was measured. Total RNA was harvested and reverse transcribed. Cells were transfected with pCR3.1 (mock) or pCR3.1/SOCS1 for 24 hours and then treated with 100 IU/mL IFN-α and 50 ng/mL IFN-λ respectively for 24 hours and the cells were collected. Cell lysates were harvested and the levels of mRNA expression of ISG15 and MxA were determined by quantitative real time PCR normalized to GAPDH. In addition, the cell were analyzed by immunoblotting with the indicated antibodies as described in Materials and Methods. The samples for Tyrosine phosphorylation of STAT1 (pSTAT1) were harvested after incubating with IFNs for 15 min. Shown is one representative Western blot out of three performed experiments. + with;—without. Data are presented as means ± SEM, n = 3. Error bars indicate standard error of mean (SEM). “*” means *p* values less than 0.05; “**” *p* values less than 0.01; “***” means *p* values less than 0.001.

We assessed whether SOCS1 over-expression could regulate IFN-induced ISRE-luciferase activity by means of ISRE reporter system. As shown in Fig 2A, IFN-α and IFN-λ induced ISRE-luciferase activities in Con1b replicon cells and over-expression of SOCS1 blocked ISRE-luciferase activity. We went on further to analyze whether SOCS1 over-expression could inhibit the expression of these ISGs. Con1b replicon cells were transfected with SOCS1-expressing vector empty vector (mock) for 24 hours and then treated with 100 IU/mL IFN-α or 50 ng/mL IFN-λ for 24 hours before ISG15 and MxA mRNA expression levels were analyzed by RT-qPCR. SOCS1 over-expression reduced mRNA levels of ISG15 induced by IFN-α (from 19.7±1.22 to 13.6±0.47-fold) and IFN-λ (from 5.57±1.02 to 1.64±0.19-fold); MxA induced by IFN-α (from 29.7±3.65 to 12.8±1.43-fold) and IFN-λ (from 11.6±1.77 to 5.47±1.21-fold), respectively (Fig 2B). To further analyze the expression levels of these ISGs at protein level, Con1b replicon cell was transfected with pCR3.1 (mock) or pCR3.1/SOCS1 for 24 hours and then treated with 100 IU/mL IFN-α and 50 ng/mL IFN-λ respectively for 24 hours and the cells were harvested and analyzed by Western blotting. Tyrosine phosphorylation of STAT1 proteins was examined after stimulation with IFN-α and IFN-λ for 15 min. As shown in Fig 2C, SOCS1 over-expression significantly reduced protein levels of ISG15 and MxA and the STAT1 tyrosine phosphorylation induced by IFN-α and IFN-λ in Con1b replicon cells. Similar results were observed in JFH1-2a replicon cell (S1 Fig).

In summary, these results indicate that SOCS1 over-expression repressed both type I and type III IFN signaling pathway, including inhibiting STAT1 phosphorylation, attenuating ISRE reporter activity, and blocking ISGs expression.

### SOCS1 over-expression blunts the antiviral effects of type I and type III IFNs

**Fig 3:**
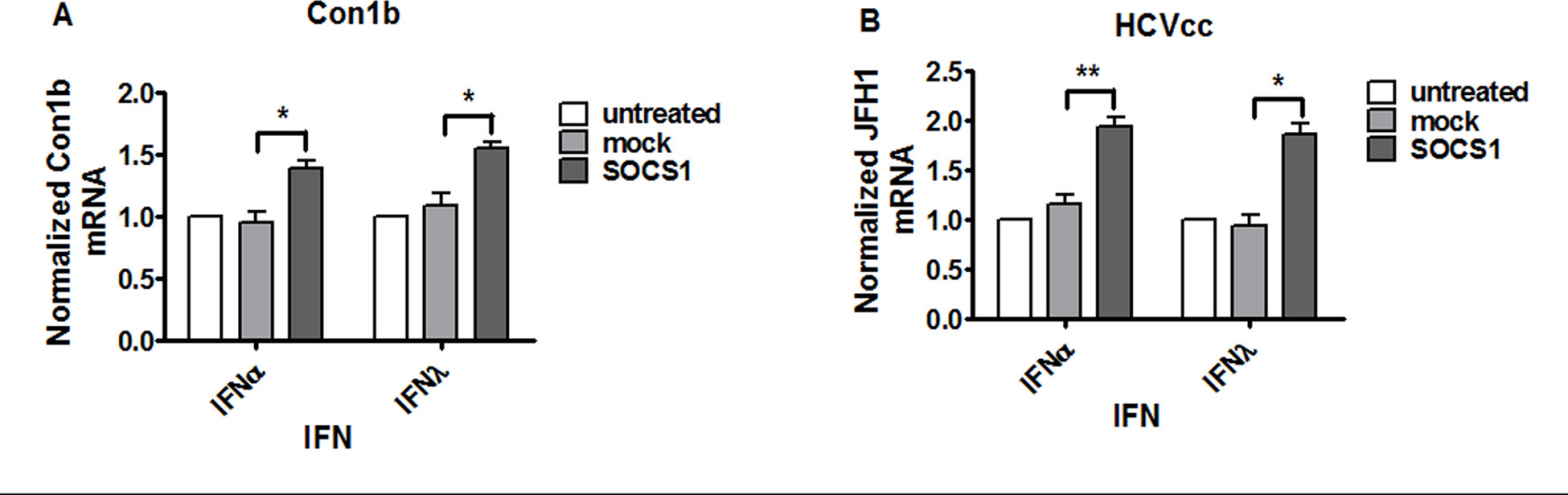
Over-expression of SOCS1 blunted both type I and type III anti-viral activity in HCV replicon cell and HCVcc system. Over-expression of SOCS1 inhibited IFN-α and IFN-λ antiviral effects in Con1b replicon (A) and JFH-1 HCVcc system (B). Cells were transfected with pCR3.1 (mock) or pCR3.1/SOCS1 for 24 hours and then treated with 100 IU/mL IFN-α and 50 ng/mL IFN-λ respectively for 24 hours and the cells were collected. Total RNA was harvested and reverse transcribed. The levels of mRNA expression of Con1b and JFH1 were determined by quantitative real time PCR normalized to GAPDH. Data are presented as means ± SEM, n = 3. Error bars indicate standard error of mean (SEM). “*” means *p* values less than 0.05; “**” *p* values less than 0.01.

To determine the role of SOCS1 over-expression in HCV RNA replication following type I or type III IFN treatment, Con1b replicon cell was transfected with 4 ug pCR3.1 (empty vector, mock) or pCR3.1/SOCS1 for 24 hours and then treated with 100 IU/mL IFN-α or 50 ng/mL IFN-λ, and HCV RNAs were quantified by quantitative RT-PCR 48 hours post transfection. Over-expression of SOCS1 promoted HCV RNA replication compared to cells transfected with pCR3.1 (empty vector, mock) (Fig 3A) in the presence of IFN-α or IFN-λ. In addition, we observed a similar stimulatory effect of SOCS1 on HCV RNA replication in HCV JFH1 cell culture system (Fig 3B) and JFH1-2a replicon cell (S2 Fig). Taken together, SOCS1 over-expression inhibits the antiviral effects of both IFNs, indicating that SOCS1 is a functional negative regulator of not only IFN-α but also IFN-λ-mediated IFN signaling pathway.

### Higher baseline SOCS1 mRNA levels in PBMCs of treatment Non-Responders than Responders

**Fig 4:**
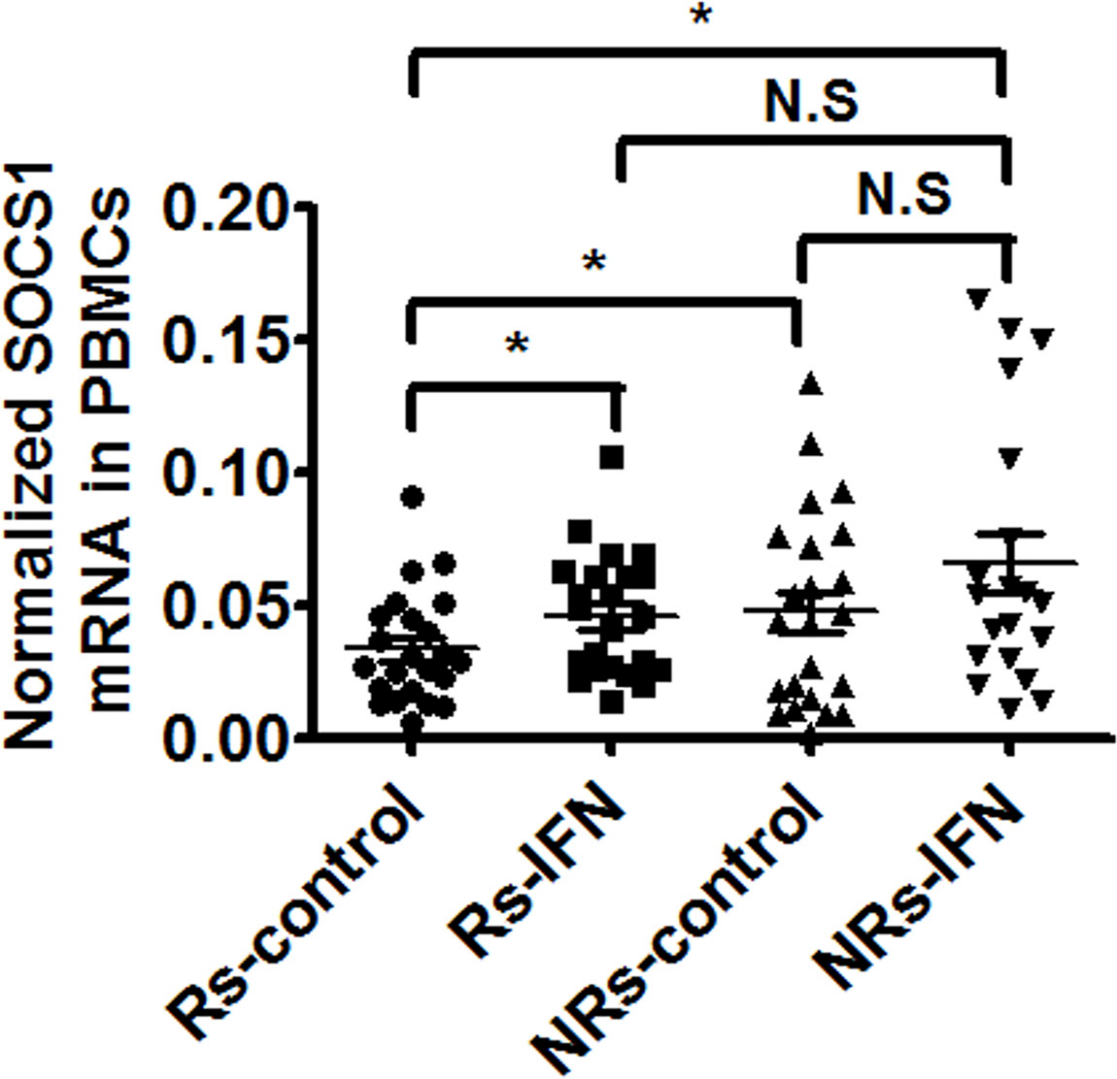
Differential mRNA expression levels of SOCS1 in PBMCs from non-responders (NRs) compared to those from patients with sustained virological response (Rs). PBMCs were cultured in vitro with/without IFN-α (500 IU/mL) treatment for 8 hours and the cells were collected. Total RNA was harvested and reverse transcribed. The levels of mRNA expression of SOCS1 were determined by quantitative real time PCR normalized to GAPDH. Data are presented as means ± SEM, n = 3. Error bars indicate standard error of mean (SEM). “*” means *p* values less than 0.05. “NS.” means no statistical significance.

To define whether SOCS1 is expressed differently in PBMCs between Rs and NRs, we quantified mRNA expression level of SOCS1 in PBMCs of all 48 patients after in vitro culture with/without IFN-α treatment for 8 hours. Although SOCS1 expression is induced by IFN-α in both responders and non-responders (Fig 4). The baseline expression level of SOCS1 is significant higher in NRs compared to Rs, which may provide a clue to treatment non-response and is potentially useful in predicting treatment outcomes in the clinic.

## Discussion

Until now the standard of care (SOC) for HCV treatment in most developing countries is weekly subcutaneous injection of pegylated IFN-α (PEG-IFN-α) combined with daily administration of ribavirin [22]. However, this IFNα-based therapy has suboptimal SVR rates in treatment resistant patients and deleterious side effects which highlights the need for alternative treatment options [23]. Type III IFN (IFN-λ) is an ideal therapeutic candidate with promising antiviral activity against HCV *in vivo* [24] and fewer side effects due to the limited receptor distribution [25]. Previous studies documented the distinct kinetics of ISG induction in hepatocytes and hepatic cell lines upon stimulation with IFN-α and IFN-λ by means of microarray technology [26–28]. IFN-λ triggered a delayed but prolonged ISG response which would meet with the expectation of a good therapeutic candidate; on the other hand, IFN-α produced a kinetic profile of anti-viral ISGs that peaked at early times of treatment and then rapidly died down which may partially explain the observed relatively poor clinical efficacy of IFN-α. Although the different activation kinetics profiles of IFN signaling and down-stream anti-viral ISG expression induced by type I (IFN-α) and type III (IFN-λ) has been demonstrated, its underlying mechanism remains unclear. In this study, we identified the differential induction pattern of SOCS1, a typical negative regulator of IFN signal pathway, may help to understand the differential induction kinetics of ISGs following type I and type III IFN stimulation. Most interestingly, we also observed a biological role for SOCS1 expression level in PBMCs that is negatively associated with treatment outcomes: higher expression levels of SOCS1 following IFN-α stimulation in treatment non-responders were observed.

First, we stimulated Huh-7.5.1 cells with IFN-α and IFN-λ to test whether SOCS1 is induced differently. While SOCS1 induction following stimulation with IFN-λ was peaked at 8 hours, IFN-α produced a prolonged SOCS1 expression, consistent with the more sustained inhibition of IFN signaling and ISG expression. Indeed, the expression of anti-HCV ISGs induced by IFN-λ was delayed but sustained (peaked at 16–24 hours but prolonged to 4–48 hours compared with 8 hours and prolonged to 1–24 hours in IFN-α treatment), which is consistent with previous studies from other groups [28, 29]. Taken together, it is reasonable to conclude that the delayed but prolonged activation of Jak/STAT signaling by IFN-λ is probably due to the faster but shorter induction of SOCS1 expression. Next, we examined the effect of SOCS1 over-expression on the anti-HCV effect of type I and type III IFNs in both HCV replicon cells and JFH1 culture system. SOCS1 over-expression significantly blunted the IFN signaling induced by both type I and type III IFNs, as shown by attenuated STAT1 phosphorylation, decreased ISRE-activity and ISG expression [30]. Lastly, we assessed the association between SOCS1 expression level in PBMCs and treatment outcomes in HCV patients. Our data indicated that although SOCS1 expression in PBMCs is induced by IFN-α in both responders and non-responders, the baseline expression level of SOCS1 is significant higher in NRs compared to Rs. Previous studies from our group indicated that differential expression level of a subset of ISGs, including ubiquitin-specific protease 18 (USP18), not only discriminates responders from non-responders of HCV patients [31–33], but also is a stronger predictor of HCV treatment outcomes than IL28B genotype [34, 35]. It has been resported that mouse USP18 interacts with IFNAR2 to negatively regulate type I IFN signaling [36–38]. However, as another negative feedback regulator of IFN signaling, whether hepatic SOCS1 expression level is associated with response to therapy in HCV infected patients remains contradictory. ImanakaK’s group reported an increased intrahepatic SOCS1 expression in non-responders [39], while Stephanie Pascarella and her colleagues demonstrated that intrahepatic mRNA levels of SOCS1 are associated with cirrhosis but do not predict virological response to therapy in patients chronically infected with HCV [19]. These opposite observations probably arise from different patient population and evaluation methods. Our data indicated that increased baseline expression levels of SOCS1 in PMBCs are correlated with treatment non-response, which are in according with Iijima’s conclusion that non-SVR patients had higher levels of SOCS1 mRNA, associated with IL28B genotype [40]. This complements our data in vitro that SOCS1 blunts both type I and type III IFN anti-HCV activity through inhibiting Jak/STAT signaling at various stages: STAT1 phosphorylation, ISRE activity and ISG expression.

In summary, in this current study, we aimed to understand the underlying molecular mechanisms why type I and type III IFNs activates IFN signaling cascade and ISG expression differently, more specifically why IFN-λ activates Jak/STAT signaling in a delayed but prolonged fashion compared to IFN-α. We found differential kinetics profiles of SOCS1 expression induced by IFN-α and IFN-λ. Faster but shorter induction of SOCS1 by IFN-λ may help explain the delayed but prolonged activation of IFN signaling and ISG expression. Furthermore, we demonstrated that the baseline SOCS1 expression in PBMCs is strongly associated with poor response to IFN-α therapy. However, the detailed molecular mechanisms underlying this association, as well as its potential diagnostic and prognostic significance, are worth further studies.

## Supporting Information

S1 Fig Over-expression of SOCS1 blocked IFN-α and IFN-λ signaling pathway in JFH1-2a replicon cell. (TIF)

S2 Fig Over-expression of SOCS1 blunted both type I and type III anti-viral activity in JFH1-2a replicon cell. (TIF)

S1 Table Primers for qRT-PCR. (DOCX)
