# Supplementary figures and images for: Type III Interferon Induces Distinct SOCS1 Expression Pattern that Contributes to Delayed but Prolonged Activation of Jak/STAT Signaling Pathway: Implications for Treatment Non-Response in HCV Patients
Source: PLoS One. 2015 Jul 20;10(7):e0133800. doi: 10.1371/journal.pone.0133800 (PMC4508043; doi:10.1371/journal.pone.0133800)

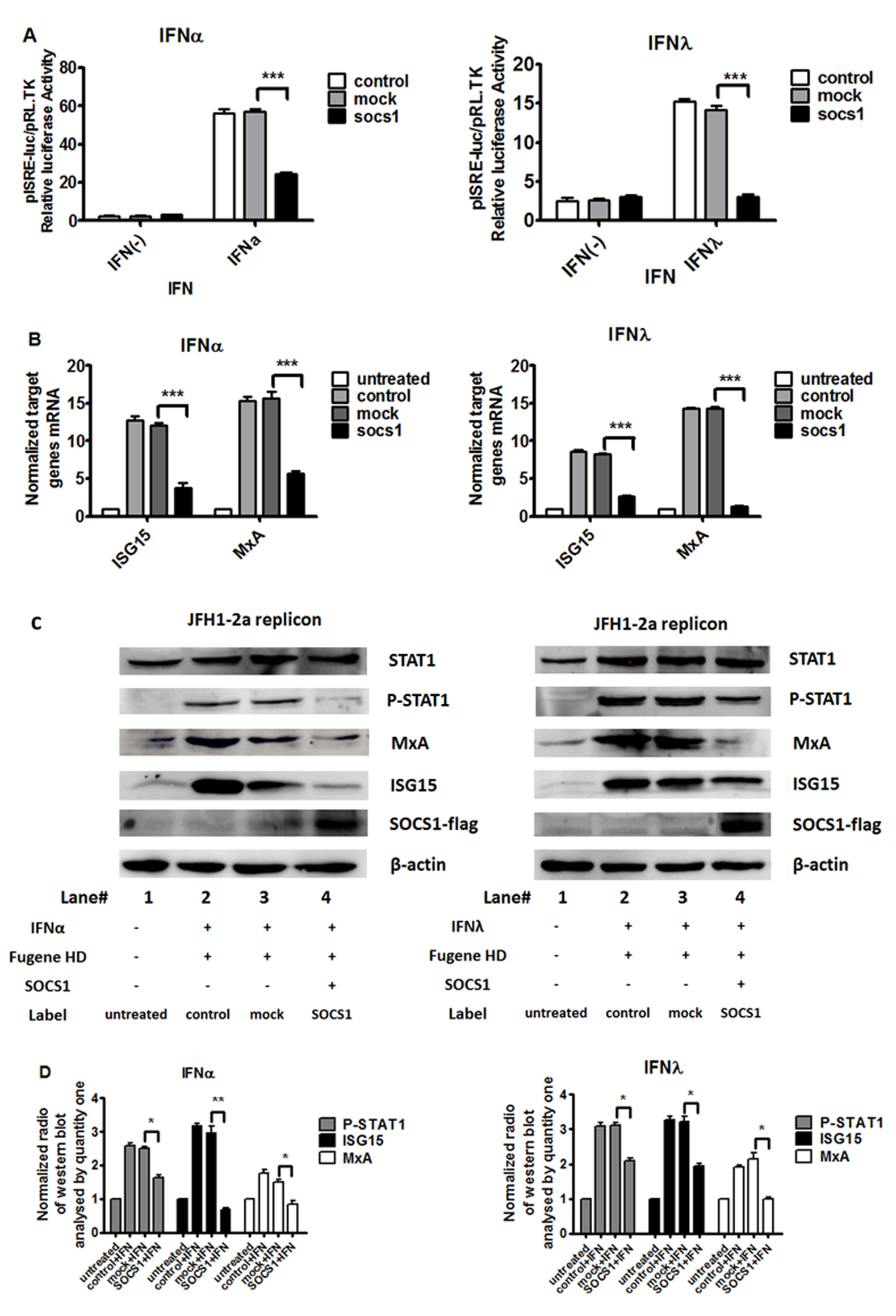

Supplement: S1 Fig — (TIF) [file pone.0133800.s001.tif]

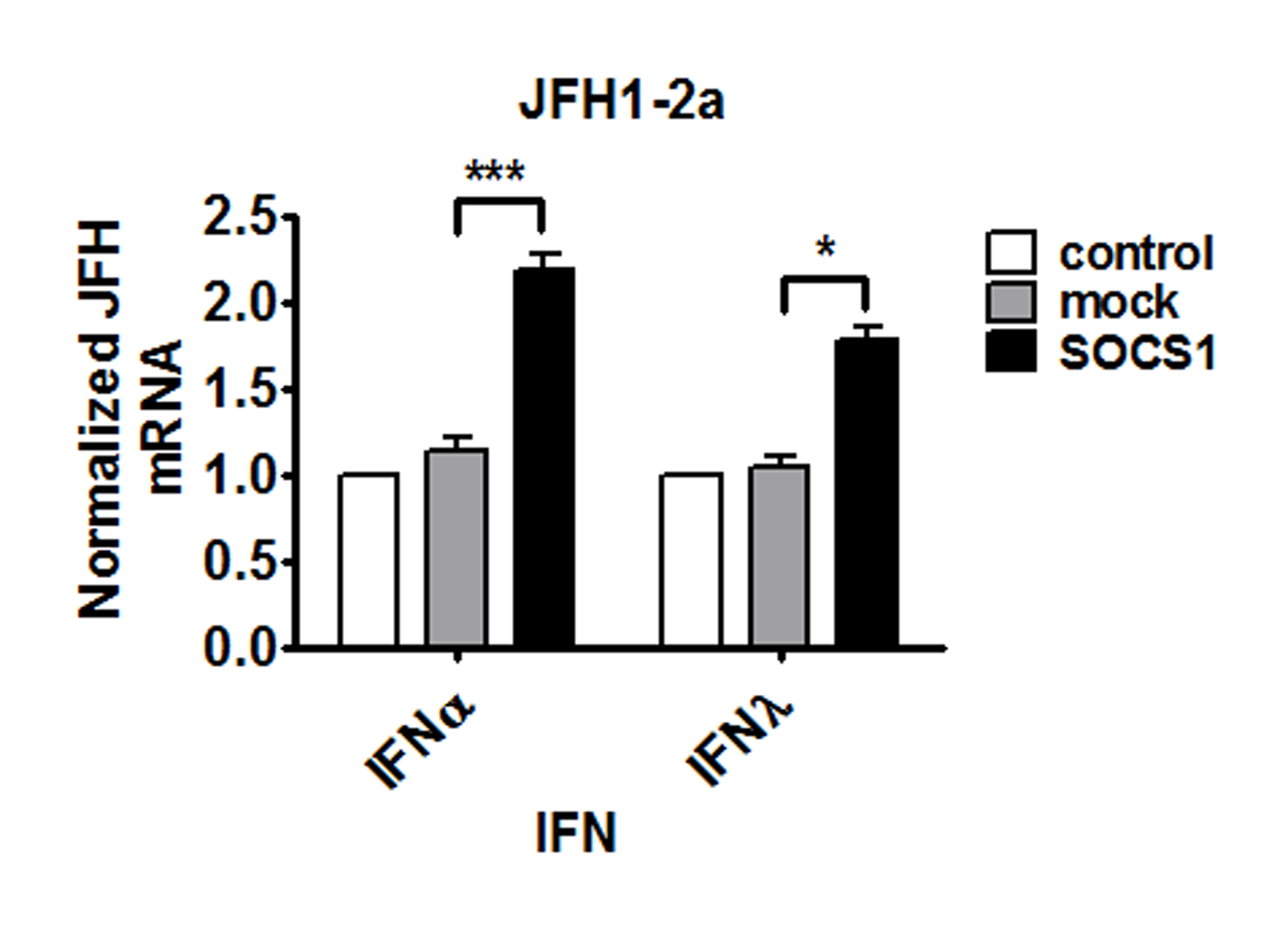

Supplement: S2 Fig — (TIF) [file pone.0133800.s002.tif]
