# Supplementary material for: Type III Interferon Induces Distinct SOCS1 Expression Pattern that Contributes to Delayed but Prolonged Activation of Jak/STAT Signaling Pathway: Implications for Treatment Non-Response in HCV Patients
Source: PLoS One. 2015 Jul 20;10(7):e0133800. doi: 10.1371/journal.pone.0133800 (PMC4508043; doi:10.1371/journal.pone.0133800)
Supplement: S1 Table — (DOCX) [file pone.0133800.s003.docx]

| **Gene** | **Sequence** |
| --- | --- |
| **SOCS1** | 5’-GGTCCCCCTGGTTGTTGTA-3’ |
|  | 5’-TAGGAGGTGCGAGTTCAGGT-3’ |
| **HCV Con1b** | 5’-GAAAGCGTCTAGCCAT-3’ |
|  | 5’-CTCGCAAGCACCCTATCAG-3’ |
| **HCV JFH-1** | 5’-GCAGAAAGCGCCTAGCCAT-3’ |
|  | 5’-CTCGCAAGCGCCCTATCAG-3’ |
| **GAPDH** | 5’-GCCTCCTGCACCACCAACTG-3’ |
|  | 5’-ACGCCTGCTTCACCACCTTC-3’ |
| **ISG15** | 5’-CGCAGATCACCCAGAAGATT-3’ |
|  | 5’-GCCCTTGTTATTCCTCACCA-3’ |
| **MxA** | 5’-GTGCATTGCAGAAGGTCAGA-3’ |
|  | 5’-CTGGTGATAGGCCATCAGGT-3’ |
